# Implications of Genetic Elements on Type 2 Diabetes Mellitus Pathogenesis and Management

**DOI:** 10.1002/edm2.70204

**Published:** 2026-03-23

**Authors:** Nokwanda N. Ngcobo, Ntethelelo H. Sibiya

**Affiliations:** ^1^ Discipline of Pharmaceutical Sciences School of Health Sciences Durban South Africa; ^2^ Pharmacology Division, Faculty of Pharmacy Rhodes University Makhanda South Africa

**Keywords:** CYP450, ethnic disparities, genetics, insulin resistance, personalised medicine, pharmacogenomics, pharmacotherapy, type 2 diabetes mellitus

## Abstract

**Background:**

Type 2 diabetes mellitus (T2DM) is a complex, multifactorial disease influenced by interactions between environmental exposures and genetic determinants. Increasing evidence highlights the important role of genetic variation in disease susceptibility, progression, and therapeutic response.

**Aim:**

This review aims to explore key genetic factors associated with T2DM risk and pathogenesis, as well as pharmacogenomic variants that influence antidiabetic therapy metabolism and clinical response.

**Results:**

Several genetic variants have been linked to altered glucose metabolism and increased susceptibility to T2DM. Notable genes include KCNJ11, HNF4A, IGF2BP2, CDKN2A/B, PPARγ, KLF14, and IRS, which influence insulin secretion, insulin signalling, and glucose regulation. Genetic polymorphisms vary across populations and may contribute to differences in disease susceptibility. In addition, variations in drug‐metabolising enzymes, particularly within the cytochrome P450 enzymes (CYP450), can influence the pharmacokinetics and pharmacodynamics of commonly prescribed antidiabetic medications. Some major CYP450 isoforms implicated in the metabolism of antidiabetic medications have been shown to exhibit polymorphisms that can alter plasma drug concentrations, ultimately leading to poor glycaemic control or heightening adverse effects.

**Conclusion:**

Therefore, understanding these genetic underpinnings is critical for advancing precision medicine approaches in diabetes care, enabling tailored pharmacotherapy and optimising treatment outcomes. By integrating genetic insights into clinical decision‐making, this paper highlights the potential of pharmacogenomics not only to identify populations at risk of developing T2DM but also to improve glycaemic control, reduce adverse drug reactions, and enhance patients' quality of life with T2DM.

## Graphical abstract

Genetic variations in T2DM, including key metabolic and insulin‐related genes, influence disease risk, insulin resistance, and drug response. Understanding these polymorphisms enables precision medicine, optimising therapy and improving glycaemic control across diverse populations.

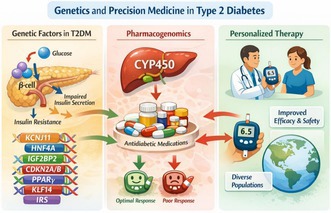

## 1 Introduction

Diabetes has been recognised as a clinical condition for more than two millennia; however, it was only in 1935 that two distinct types of diabetes were formally described. Although both types are characterised by chronic hyperglycaemia, type 1 diabetes is an autoimmune disorder resulting in the destruction of insulin‐producing pancreatic β‐cells [1]. In contrast, type 2 diabetes mellitus (T2DM) develops when insulin secretion fails to adequately compensate for increasing insulin resistance in key metabolic tissues, including skeletal muscle, liver, and adipose tissue [2].

T2DM is a complex disorder driven by interactions between genetic susceptibility and environmental and lifestyle factors such as obesity, poor diet, physical inactivity, and psychosocial stress [3]. These environmental exposures do not affect all individuals equally, highlighting the role of genetic predisposition in disease development and therapeutic response. Although the hereditary nature of T2DM has long been recognised, advances in human genetics during the 1980s enabled the identification of specific loci contributing to disease risk [4].

The primary goal of pharmacological management in T2DM is sustained glycaemic control. Current therapeutic options include insulin, biguanides, thiazolidinediones, DPP‐4 inhibitors, GLP‐1 analogues, and GLP‐1 receptor antagonists, each acting through distinct mechanisms. Notably, several of these agents are substrates for, or modulators of, cytochrome P450 (CYP450) enzymes, introducing variability in drug response and safety. This article explores the genetic underpinnings of T2DM, including CYP450 polymorphisms and their implications for therapeutic efficacy. With a deeper understanding of this complex genetic phenomenon, scientists and healthcare professionals can better predict disease risk and optimise treatment strategies for individuals at risk of or living with T2DM.

## 2 Type 2 Diabetes Genetic Blueprint

T2DM is a multifactorial disease influenced by both genetic and environmental determinants. Traditionally, prevention and management strategies have focused on modifiable lifestyle factors, while clinicians assess measurable risk indicators such as body weight, blood pressure, glycaemic status, and lipid profiles. However, family history and racial or ethnic background are also well‐established risk factors, accentuating the importance of genetic susceptibility. Estimates suggest that genetic factors account for approximately 20%–80% of interindividual variability in T2DM risk [4].

**FIGURE 1:**
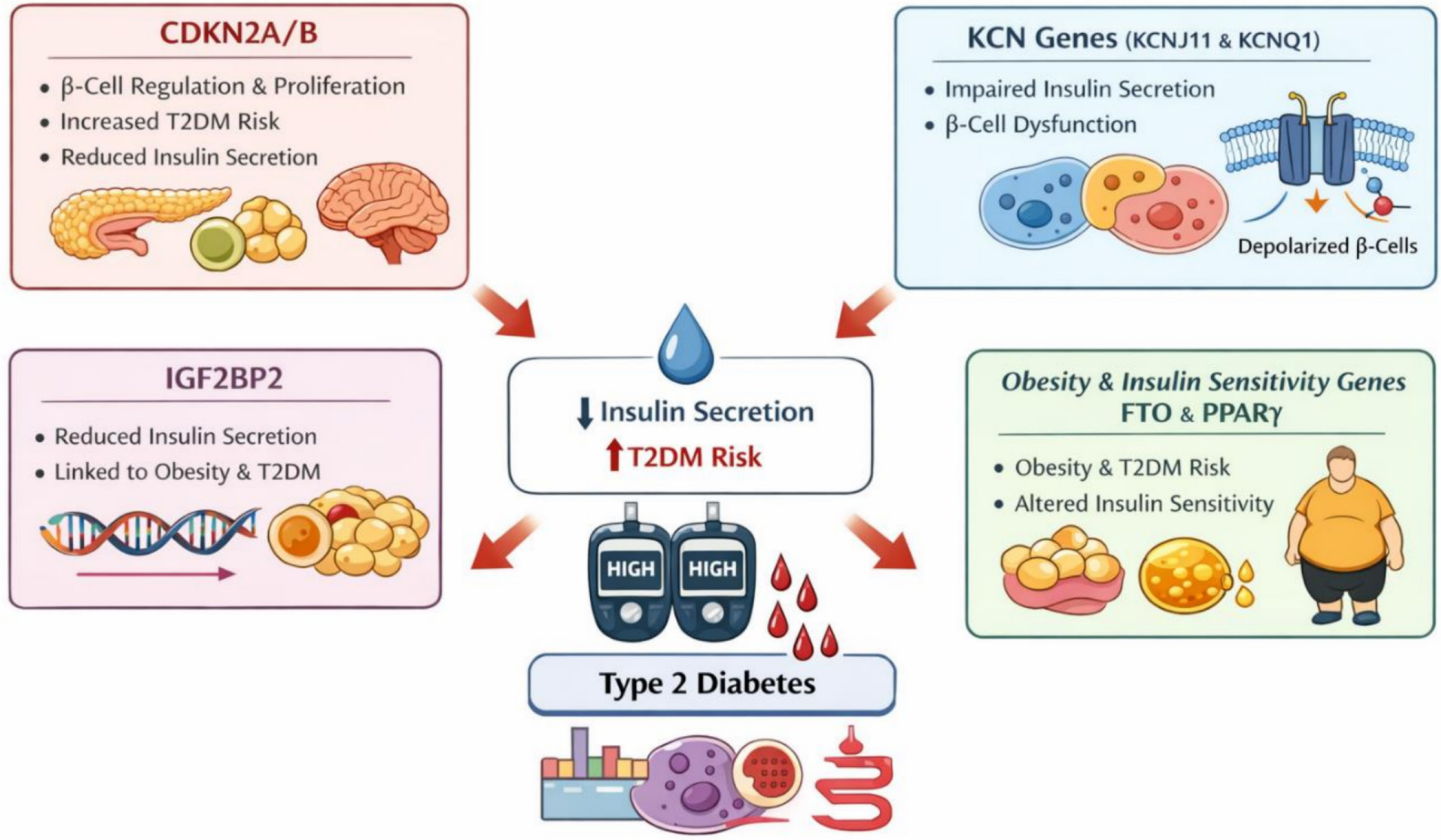
Overview of insulin secretion‐ and obesity‐related genes implicated in type 2 diabetes.

Genetic variation (see Figure 1) is a major contributor to observed ethnic disparities in T2DM prevalence, although most genetic studies have been conducted in populations from the USA, Europe, and East Asia [5]. Consequently, genetic risk variants relevant to underrepresented populations may remain unidentified. Environmental exposures also modify genetic risk; for example, arsenic exposure has been associated with increased diabetes prevalence in several regions worldwide [6, 7, 8].

Studies in admixed populations have demonstrated associations between genetic ancestry and T2DM risk. In Mexican‐American and Pima Indian populations, greater American Indian ancestry has been linked to higher diabetes prevalence [9]. The use of ancestry‐informative markers has strengthened these observations, although socioeconomic status often attenuates these associations, indicating potential confounding and emphasising the complex interplay between genetic ancestry and environmental factors in T2DM risk. In contrast, in African‐American populations, higher African ancestry remains independently associated with increased diabetes risk even after socioeconomic adjustment [10]. These findings illustrate the complex interplay between genetics, environment, and social determinants of health.

Despite substantial progress, the precise genetic blueprint underlying T2DM remains incompletely defined. Monogenic forms of diabetes, such as maturity‐onset diabetes of the young (MODY), account for approximately 5% or fewer cases of non‐autoimmune diabetes and result from mutations in highly penetrant genes, including HNF1A and GCK [11, 12]. Although clinically distinct from T2DM, variants in these genes may also contribute to polygenic diabetes risk [11].

Genome‐wide association studies (GWAS) have identified over 200 genetic variants associated with T2DM across diverse populations [13, 14]. Large multi‐ancestry analyses have further demonstrated substantial genetic and metabolic heterogeneity, with distinct clusters of variants influencing β‐cell function, insulin resistance, adiposity, and cardiometabolic traits [15]. Importantly, variation in allelic effect sizes across populations is driven less by differences in allele frequencies and more by gene–environment interactions and study design factors [16, 17].

While most T2DM‐associated variants confer modest effects, rare population‐specific variants may substantially increase disease risk in certain groups. Examples include HNF1A variants in Oji‐Cree populations and TBC1D4 variants among Inuit populations [9, 18, 19]. Several genes involved in insulin secretion and action play critical roles in the pathogenesis of T2DM. Variants in KCNJ11, which encodes a component of the ATP‐sensitive potassium channel, impair β‐cell function and insulin release [8, 20]. Genes influencing insulin sensitivity and lipid metabolism, including PPARγ, KLF14, and IRS‐1, are also strongly implicated. Polymorphisms in KLF14 are associated with dyslipidaemia and insulin resistance [21, 22], while the IRS‐1 G972R variant disrupts insulin signalling [23].

Ethnic variability and epigenetic regulation further modulate the effects of these polymorphisms [21]. Variants in DUSP9, which regulates MAPK signalling, have been linked to altered insulin sensitivity, particularly in females [24, 25]. The FTO rs9939609 polymorphism influences obesity and insulin resistance by altering energy balance [26]. Additional genes affecting β‐cell function include HNF4A, IGF2BP2, and CDKN2A/B, all of which contribute to impaired insulin secretion and increased diabetes susceptibility [24, 27, 28]. Among the most robustly replicated genetic risk factors for T2DM is the TCF7L2 rs7903146 variant, which is associated with reduced insulin secretion through altered β‐cell and Wnt signalling pathways [29]. The following sections examine these genetic polymorphisms in greater detail and explore their roles in the pathogenesis and clinical heterogeneity of T2DM.

## 3 Insulin Secretion‐Associated Genes

### 3.1 KCNJ11 Gene

Regarding the KCNJ11 gene, the rs5219 variant allele may reduce channel sensitivity to ATP and alter the charge of the ATP‐binding region (see Table 1) [42]. A recent meta‐analysis and case–control study established a strong correlation between rs5219 polymorphisms and T2DM susceptibility in both East Asian and Caucasian populations [43, 44]. Additionally, Kir6.2, the protein encoded by KCNJ11, is expressed in neurons, the brain, and muscle tissues, further implicating its role in glucose metabolism and insulin regulation [45]. Li et al. identified significant associations between ADIPOQ gene polymorphisms (rs1501299, rs182052, and rs7627128) and T2DM in a Chinese population [46]. Additionally, a haplotype‐based case–control study examining the association between ADIPOQ and T2DM found that the A–A–T haplotype was associated with increased risk, whereas the G–A–T haplotype was associated with reduced risk [46]. Thus, further research is required to elucidate these genetic contributions and their interactions with environmental factors.

**TABLE 1:** Key genetic polymorphisms associated with type 2 diabetes mellitus: mechanisms and population‐based evidence.

Gene	Polymorphisms	Function	Association with T2DM	Population/ethnic findings	Estimated OR (95% CI)
CDKN2A/B	Various including p16INK4A/p14ARF, *rs10811661*	Regulates β‐cell mass, insulin secretion, apoptosis, and cell cycle.	Reduced insulin secretion. Affects GSIS and insulinogenic index	Asian European	Modest ORs (~1.2–1.3) historically [30, 31]
KCNJ11	rs5219	Encodes Kir6.2, part of the KATP channel involved in insulin secretion.	Impaired insulin secretion	East Asian Caucasian	~1.15–1.6 [32, 33]
KCNQ1	rs2283228, rs2237895	Regulates insulin exocytosis and glucose homeostasis.	β‐cell function dysfunction	Asian populations	~1.5–1.6 [34]
IGF2BP2	rs4402960, rs1470579	Regulates β‐cell function and interacts with obesity‐related mechanisms.	β‐cell function dysfunction Obesity	Multi ethnic	~1.3–1.7 depending on genetic model [35]
TCF7L2	rs7903146	Affects β‐cell survival, insulin secretion, and transcriptional regulation.	Reduced insulin secretion	Multi ethnic	~> 1.0–2.0 depending on population [36, 37]
PPARγ	Pro12Ala (rs1801282)	Regulates adipocyte differentiation, lipid metabolism, and insulin sensitivity.	Ethnic‐dependent effects. Ala12 allele is protective in Caucasians (↑ sensitivity) Possibly harmful in Indians (↑ T2DM risk)	Caucasians South Indians	~0.8–0.9 (protective) [38]
DUSP9	rs5945326	Inactivates ERK/JNK; regulates insulin signalling and cell cycle.	Insulin resistance	European Japanese	~1.10–1.40 [39, 40]
FTO	rs1558902	Associated with body weight and BMI regulation.	Obesity mediated T2DM risk	Multi ethnic	~1.4–1.7 [34, 41]

Mutations in KCN genes have been linked to the onset of diabetes. Variants in the KCNQ1 gene have been associated with reduced insulin exocytosis in response to depolarization [24, 47]. The C allele of the rs2283228 variant has been linked to elevated fasting glucose levels and impaired β‐cell function in Asian populations [24]. Additionally, research has shown that most of the genomic loci identified so far are associated with β‐cell dysfunction in individuals with T2DM [48, 49]. KCNQ1 is expressed in pancreatic islets and plays a crucial role in maintaining glucose homeostasis by regulating insulin secretion [50]. The KCNQ1 protein has also been detected in insulin‐secreting INS‐1 cells [24]. Furthermore, studies have reported that the C allele of the KCNQ1 intronic rs2237895 variant is associated with a lower risk of abdominal obesity in individuals with T2DM. Specifically, this allele has been associated with reduced BMI and waist circumference in a Chinese population [51].

### 3.2 IGF2BP2

Elssaig et al. conducted a population‐based case–control study and reported significant associations between the IGF2BP2 rs1470579 and rs4402960 polymorphisms and increased T2DM risk in a Saudi Arabian cohort, alongside a proposed genetic screening model that requires further validation [52]. Another study found that T2DM patients carrying the C allele of rs1470579 had higher fasting plasma glucose, total cholesterol, and postprandial serum insulin levels (see Table 1) than those with the AA genotype [53]. IGF2BP2 polymorphisms are implicated in the regulation of pancreatic β‐cell function [54]. Additionally, research has shown a strong association between IGF2BP2 and obesity. Given the established connection between obesity and T2DM, it is hypothesized that obesity may influence the association between IGF2BP2 and T2DM, a phenomenon referred to as the interplay between IGF2BP2, obesity, and T2DM [53, 55]. Supporting this hypothesis, Cao et al. reported that IGF2BP2 expression levels in adipose tissue were more than twice as high in T2DM patients compared to healthy individuals [56]. Further studies in Mexican American and Canadian Caucasian populations have linked IGF2BP2 to visceral and abdominal fat accumulation, suggesting a potential role in insulin resistance [24, 57].

### 3.3 TCF7L2 (Transcription Factor 7‐Like 2)

Variants in TCF7L2, particularly rs7903146, are among the most consistently replicated genetic risk factors for T2DM and are strongly associated with impaired β‐cell function and reduced insulin secretion [58]. However, despite its robust association with disease susceptibility across multiple ethnic groups, knowledge of the TCF7L2 genotype does not currently guide routine clinical decision‐making. At present, its primary clinical relevance lies in risk stratification and prognostication rather than treatment selection [59].

Emerging evidence suggests that carriers of the rs7903146 risk allele may exhibit reduced responsiveness to insulin secretagogues, particularly sulfonylureas, and an earlier need for insulin therapy [60]. Nevertheless, these observations have not yet been translated into formal therapeutic guidelines. Consequently, TCF7L2 genotyping is best viewed as a marker of β‐cell vulnerability that may inform future precision medicine approaches, rather than a determinant of current therapeutic choices [58].

### 3.4 CDKN2A/B

Polymorphisms in the CDKN2A/B gene influence metabolic health by affecting proteins involved in β‐cell mass regulation, insulin secretion, and cell proliferation [61]. Studies conducted in both Asia and Europe have consistently confirmed an association between CDKN2A/B variants and T2DM risk. CDKN2A/B is highly expressed in adipocytes, pancreatic islet cells, and brain cells. As tumour suppressor genes, CDKN2A and CDKN2B play a critical role in apoptosis, tumorigenesis, and cellular proliferation [24, 62, 63].

In a study of Europeans who underwent a hyperglycaemic clamp, the CDKN2A/B gene alone was not independently associated with β‐cell function [64]. Each additional risk allele in this group was associated with a 5% reduction in glucose‐stimulated insulin secretion (GSIS), though insulin sensitivity remained unaffected [30]. This negative impact on insulin secretion was observed in both individuals with normal and impaired glucose tolerance, indicating that the influence of CDKN2A/B may occur before overt metabolic dysfunction develops (refer to Table 1).

Further support comes from a large meta‐analysis study, which identified associations between CDKN2A/B and measures such as the insulinogenic index, acute insulin response, fasting glucose, and HOMA‐B, but found no association with insulin sensitivity index, fasting insulin, fasting proinsulin, or HOMA‐IR [30, 65, 66]. Additionally, a related investigation into the effects of CDKN2A/B genotypes on islet function, both in vivo and ex vivo, showed reduced insulin secretion and a lower disposition index in vivo, without changes in insulin sensitivity or glucagon secretion. No significant effects on ex vivo islet function were detected. Interestingly, individuals with lower p16INK4A activity or p16INK4A/p14ARF exhibited increased basal and stimulated insulin secretion [30, 67, 68].

However, similar to TCF7L2, individual CDKN2A/B variants currently have limited direct clinical utility when considered in isolation. Hyperglycaemic clamp studies and large meta‐analyses indicate that these variants primarily affect insulin secretory capacity rather than insulin sensitivity, suggesting a role in early β‐cell failure rather than overt metabolic dysregulation. Clinically, CDKN2A/B variants are most informative when incorporated into multi‐locus genetic risk scores, where cumulative risk alleles have been shown to predict progressive decline in β‐cell function. Such composite profiles may eventually help identify individuals at risk of earlier disease onset or rapid progression. However, at present, CDKN2A/B genotyping does not directly alter therapeutic selection or dosing strategies and remains a research‐oriented tool with potential future application in preventive and prognostic frameworks.

## 4 Insulin Sensitivity and Obesity‐Related Genes

### 4.1 FTO Gene

Numerous studies have established a strong association between the FTO gene and obesity, a major risk factor for T2DM [41]. Individuals with T2DM, particularly in East Asian populations, often reach their peak lifetime BMI at or before disease onset, with post‐diagnosis BMI influenced by lifestyle modification and antidiabetic therapy [69]. Genetic association studies have demonstrated significant relationships between FTO single‐nucleotide polymorphisms (SNPs) and BMI, including sex‐specific effects [70]. The rs1558902 variant has been associated with increased T2DM incidence, with effects persisting after adjustment for age and BMI, while rs9939609 may influence T2DM risk partly independent of adiposity [41].

Mechanistically, increased FTO expression has been linked to reduced mitochondrial oxidative capacity, enhanced oxidative stress, and lipid accumulation. These alterations promote de novo lipogenesis, suppress lipolysis and fatty acid oxidation, and increase hepatic gluconeogenesis, collectively contributing to triglyceride accumulation and dysregulated glucose homeostasis (Table 1) [71].

### 4.2 PPR‐Gamma Polymorphism

PPARγ is a nuclear receptor that plays a crucial role in adipocyte differentiation, lipid metabolism, and insulin sensitivity [72]. The Pro12Ala polymorphism (rs1801282) in the PPARγ2 gene has been extensively studied for its association with T2DM across various populations [73]. Functionally, the Ala12 variant reduces PPARγ‐mediated transactivation of PPARγ‐responsive promoters, resulting in lower transcriptional activity than the Pro12 isoform. This altered activity is thought to modulate adipocyte function and systemic insulin sensitivity [73]. Numerous case–control studies have shown that the Pro12Ala (Ala12) variant is associated with a reduced risk of T2DM in East Asian populations (including Japanese), Greater Middle Eastern populations, and several European groups, including Finnish, Czech, and White Scottish populations [38]. In contrast, other studies have reported the PPARG Pro12Ala variant as a potential risk allele, conferring increased susceptibility to T2DM in Russian, South Asian (Kashmiri), and mixed‐ancestry South African populations [38].

In several populations, the Ala12 allele has been consistently associated with enhanced insulin sensitivity, lower BMI, and a reduced risk of T2DM, suggesting a protective metabolic phenotype [74, 75]. These findings are biologically coherent with reduced PPARγ activity, which limits excessive adipogenesis and ectopic fat accumulation. Moreover, the observed improved insulin sensitivity may partly explain interindividual variability in response to thiazolidinediones, for which PPARγ is a key therapeutic target [75]. However, this protective effect is not universally observed. In South Indian populations, the rs1801282 has been associated with increased susceptibility to T2DM and diabetic nephropathy, highlighting important ethnic‐specific differences [76]. Asian Indians, who are globally recognised as a high‐risk group for T2DM, typically exhibit greater central adiposity, higher insulin resistance, and increased cardiometabolic risk despite lower BMI thresholds compared to other ethnicities [77]. In this context, reduced PPARγ activity may exacerbate metabolic dysfunction rather than confer protection, particularly when combined with adverse environmental exposures such as high‐carbohydrate diets and sedentary lifestyles.

These conflicting findings suggest that the clinical impact of rs1801282 is highly context dependent, influenced by gene–gene interactions, environmental factors, and baseline metabolic profiles. While some studies report robust associations, others are limited by small sample sizes, population stratification, or inadequate adjustment for confounders such as obesity and medication use. Collectively, the evidence highlights that PPARγ Pro12Ala is not a universal risk or protective marker, but rather a modifier of metabolic risk whose effects vary across populations. This has important translational implications, emphasising the need for ethnicity‐informed genetic risk stratification and personalised approaches to T2DM prevention and therapy.

### 4.3 DUSP9 Gene

DUSP9 encodes a dual‐specificity phosphatase that negatively regulates mitogen‐activated protein kinases, particularly ERK and JNK, in insulin signalling. By attenuating stress‐activated kinase signalling, DUSP9 plays a protective role against insulin resistance and has been implicated in glucose homeostasis [25]. Mechanistically, impaired DUSP9 activity may prolong inflammatory and stress‐related signalling, thereby contributing to defective insulin action [25, 78].

The association between DUSP9 and T2DM was first identified in genome‐wide association studies in European populations, where the rs5945326 variant was linked to increased diabetes risk [24, 79]. Subsequent replication in Japanese cohorts strengthened the evidence for a conserved role of DUSP9 across ethnically distinct populations [80]. Furthermore, studies in Pakistani populations demonstrated that SNPs in or near DUSP9 were associated with T2DM with effect sizes comparable to those observed in European cohorts, suggesting a relatively consistent genetic influence (refer to Table 1).

Despite these consistent associations, the translational relevance of DUSP9 variants remains incompletely defined. Most studies rely on statistical associations rather than direct functional validation, and the tissue‐specific effects of DUSP9 on insulin signalling remain incompletely elucidated. Additionally, effect sizes are modest, indicating that DUSP9 polymorphisms likely contribute to T2DM risk in combination with other genetic and environmental factors rather than acting as independent predictors. Nevertheless, given its mechanistic role in stress‐induced insulin resistance, DUSP9 represents a potential therapeutic target and risk stratification tool, particularly in populations with heightened inflammatory or metabolic stress.

## 5 Impact of CYP450 Polymorphisms on Therapeutic Outcomes in T2DM Management

**FIGURE 2:**
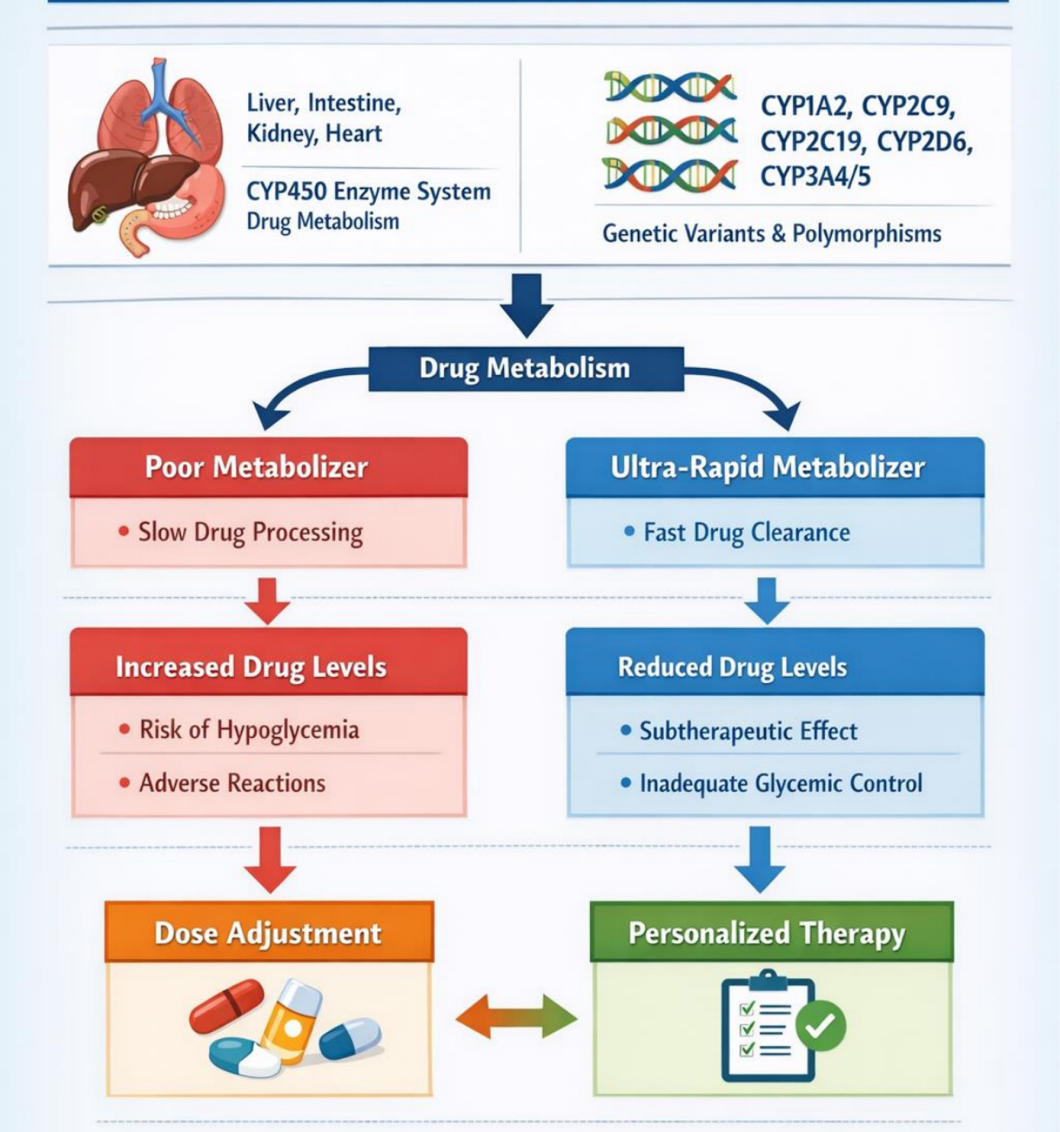
CYP450 genetic variability and therapeutic response in type 2 diabetes.

The CYP450 enzyme system comprises a group of enzymes that metabolise various drugs. While these enzymes are predominantly located in the liver, they are also found in extrahepatic tissues, including the small intestine, lungs, kidneys, and heart [81]. In hepatic metabolism, they play a crucial role in first‐pass metabolism, contributing to the greater pharmacokinetic variability observed in orally administered drugs compared to intravenous ones [82]. Among the numerous CYP450 isoforms, *CYP1A2, CYP2C9, CYP2C19, CYP2D6*, and *CYP3A4/5* are the most extensively studied, collectively metabolising over 90% of substrate drugs and significantly impacting their pharmacokinetic and pharmacodynamic properties (refer to Figure 2).

CYP450 systems play a crucial role in influencing the therapeutic efficacy of various antidiabetic medications, including sulfonylureas, metformin, and thiazolidinediones, by affecting their metabolism and clearance [81]. The CYP450 enzyme family comprises a wide range of enzymes that exhibit significant genetic variability, leading to inter‐individual differences in drug response and adverse effects (see Figure 2). Understanding these polymorphisms provides critical insights into personalised medicine and into optimising diabetes management.

### 5.1 CYP2C92 and CYP2C93

Several antidiabetic drugs used in T2DM are metabolised by CYP450 enzymes, particularly *CYP2C9* and *CYP2C8*. Genetic polymorphisms in these enzymes, such as CYP2C92, *CYP2C9*3, *CYP2C83*, and *CYP2C84*, can reduce enzymatic activity, slowing drug metabolism and increasing plasma drug levels, thereby increasing the risk of adverse effects such as hypoglycaemia, thus leading to variations in drug efficacy and safety profiles [83]. *CYP2C9* metabolises sulfonylureas (glipizide, glimepiride, glyburide) with reduced‐function variants resulting in prolonged drug action and a heightened risk of hypoglycaemia, especially with glyburide and glimepiride [84, 85]. *CYP2C8* is the main enzyme metabolising thiazolidinediones (pioglitazone and rosiglitazone) and contributes to the metabolism of repaglinide [86]. Impaired *CYP2C8* activity, either genetically or due to drug interactions, can elevate drug levels and enhance hypoglycaemic risk. Therefore, these polymorphisms may affect the metabolism of key oral antidiabetic drugs. Patients carrying these variants may require dose adjustments to prevent toxicity and optimise therapeutic outcomes [87, 88].

The findings by Saberi et al. indicated that the wild‐type allele, *CYP2C91*, was the most prevalent, with a frequency of 0.80, whereas the variant alleles *CYP2C92* and *CYP2C93* occurred at frequencies of 0.15 and 0.05, respectively. No significant associations were observed between *CYP2C9* genotypes and the patients' clinical or biochemical parameters. However, carriers of the *CYP2C93* allele exhibited the highest plasma concentrations of sulfonylureas (glibenclamide and gliclazide). Additionally, 75.9% of individuals with variant genotypes reported experiencing hypoglycaemic events [89]. More recent clinical research and meta‐analyses indicate that individuals with *CYP2C9* variant alleles tend to have an increased incidence of sulfonylurea‐induced hypoglycaemia and, in some cases, display greater HbA1c reductions at equivalent doses, consistent with slower metabolism and higher drug exposure [90]. Furthermore, Yang et al. reported that the *CYP2C93* allele in particular remains strongly associated with substantially reduced metabolic activity, with carriers showing significantly lower enzyme clearance and higher sulfonylurea plasma levels (e.g., glyburide and glimepiride) compared to wild types, an effect observed in vitro and supported by clinical pharmacokinetic trends [91].

Similarly, thiazolidinediones (TZDs) like pioglitazone and rosiglitazone, metabolised by *CYP2C8*, may require dose escalation in ultra‐rapid metabolizers due to their reduced plasma exposure [86, 92]. Meta‐analytic data show that CYP2C8*3 has one of its highest frequencies in individuals of European ancestry (~10%–12%), consistent with earlier reports of ~12%. This allele is much less common in Asian and African groups [93]. This gain‐of‐function variant has been associated with a modest increase in the metabolic clearance of TZDs. In a study by Yin et al., it was found that individuals carrying the CYP2C9*3 variant showed significantly reduced plasma exposure to pioglitazone, about 50%–77% lower in AUC, C_max_, and T_max_, along with higher metabolite‐to‐parent ratios [94]. This indicates that pioglitazone is cleared more rapidly and metabolised more extensively in these individuals. The findings suggest that CYP2C9*3 may contribute to an ultrarapid metabolizer phenotype, particularly in the Chinese population, which could lead to subtherapeutic drug levels and reduced glucose control **i**n diabetic patients.

On the other hand, poor or intermediate metabolizers exhibit slower drug metabolism, leading to increased drug accumulation and a heightened risk of adverse effects. Sulfonylureas, particularly those metabolised by *CYP2C9*, pose a significant risk of hypoglycaemia in poor metabolisers due to prolonged drug exposure, warranting a dose reduction [84]. A study by Yee et al. found that individuals carrying *CYP2C9* variant alleles (2 and 3) had a higher overall risk of hypoglycaemia than wild‐type homozygotes (OR ≈ 1.24). Specifically, the CYP2C9*2 allele was significantly linked to increased hypoglycaemia risk (OR ≈ 1.85), while the CYP2C9*3 allele showed a trend toward increased risk, but this did not reach statistical significance in the pooled analysis [90]. Based on these findings, *CYP2C9* genotyping could serve as a valuable tool for identifying patients at increased risk of hypoglycaemia during sulfonylurea therapy in T2DM, potentially guiding more personalised and safer treatment strategies.

In relation to the impact of *CYP2C9* genotype on sulfonylurea pharmacokinetics, historical studies of Lee et al. reported the AUC to be increased by 1.5‐fold in *CYP2C91/2* carriers and 1.9‐fold in *CYP2C91/3* carriers relative to wild‐type individuals [95]. These differences are attributed to the decreased metabolic capacity of mutant *CYP2C9* alleles. Supporting this, in vitro studies have demonstrated that the CYP2C*92 and *3 variants significantly impair the intrinsic clearance of sulfonylureas such as glimepiride and gliclazide [91]. Clinically, this reduced metabolic activity leads to a stronger glucose‐lowering effect in individuals with variant alleles.

In parallel, pharmacometabolomics studies in patients treated with sulfonylureas have identified distinct metabolic profiles associated with drug response, highlighting the potential of metabolomics to elucidate inter‐individual variability in sulfonylurea effectiveness and adverse risk [96]. These lines of evidence support moving beyond pharmacokinetics alone toward integrated pharmacogenetic and pharmacometabolomic approaches that can inform precision dosing and risk mitigation for T2DM therapies. Recent pharmacometabolomics studies have further strengthened the clinical relevance of *CYP2C9*–sulfonylurea interactions by linking *CYP2C9* genotype to circulating sulfonylurea metabolites, endogenous glucose‐regulatory pathways, and hypoglycaemia risk profiles [96, 97]. These studies provide mechanistic insights beyond conventional pharmacokinetic measures, demonstrating how reduced‐function *CYP2C9* alleles are associated with prolonged parent drug exposure and distinct metabolic signatures. Importantly, this emerging evidence has been incorporated into contemporary pharmacogenetic guidance. Among carriers of *CYP2C9* variant alleles, the primary clinical effect observed was enhanced therapeutic efficacy of certain sulfonylureas, including glibenclamide, gliclazide, and tolbutamide. As these findings consistently indicated a beneficial effect without evidence of clinically relevant harm, the Dutch Pharmacogenetics Working Group (DPWG) concluded that no therapeutic intervention or dose adjustment is warranted for these gene–drug interactions [98]. For glimepiride, studies reported increased efficacy in *CYP2C9* variant carriers, while one study identified an elevated risk of hypoglycaemia [99]. Given that insufficient glycaemic response is more frequently encountered in sulfonylurea therapy than hypoglycaemic events, the DPWG determined that the improvement in efficacy outweighed the potential risk. Consequently, no specific clinical action is recommended for the *CYP2C9*–glimepiride interaction [98].

### 5.2 CYP3A4 and CYP3A5

*CYP3A4* and *CYP3A5* are essential enzymes within the CYP450 family, responsible for metabolising a broad range of drugs, including certain antidiabetic medications such as repaglinide and saxagliptin. Polymorphisms in these genes lead to interindividual and interethnic variability in drug metabolism, affecting therapeutic efficacy, dosing requirements, and the risk of adverse effects in diabetic patients. The most well‐characterised polymorphisms include *CYP3A4*22 (rs35599367), associated with reduced enzyme activity, and *CYP3A5*3 (rs776746), *CYP3A5*6, and *CYP3A5*7, which result in a loss of *CYP3A5* enzyme function [100, 101].

Polymorphisms in these enzymes, such as the CYP3A5*3 allele, can alter drug clearance rates and therapeutic outcomes. Saxagliptin and linagliptin, commonly prescribed DPP‐4 inhibitors, are metabolised by *CYP3A4/5*. Variants in these enzymes, such as CYP3A5*3, can reduce drug clearance, leading to higher drug levels and an increased risk of side effects, such as nausea or headache [102, 103, 104]. For example, individuals expressing lower *CYP3A5* activity may experience prolonged drug action, necessitating dose modifications to avoid toxicity [101, 102]. Genetic testing for *CYP3A5* activity in individuals receiving DPP‐4 inhibitors can optimise dosing strategies, particularly in populations with high variant frequencies (e.g., African ancestry) [105, 106, 107].

The distribution of *CYP3A4/5* polymorphisms varies significantly across ethnic populations, leading to differences in drug response. African populations exhibit a higher prevalence of the *CYP3A5*1 allele, leading to increased enzyme activity [107]. As a consequence, antidiabetic drugs metabolised by *CYP3A4/5*, such as repaglinide and saxagliptin, are metabolised more rapidly, potentially leading to lower drug levels and reduced therapeutic efficacy [103, 108]. In these patients, higher doses or alternative medications may be necessary to achieve optimal glycaemic control.

Individuals with the *CYP3A51/1* genotype are considered fast metabolizers and typically require higher drug doses to achieve therapeutic blood concentrations. In contrast, *CYP3A53/3* carriers are poor metabolizers, often requiring lower doses to reach target drug levels, whereas those with the *CYP3A51/3* genotype exhibit intermediate metabolic activity. Among the Western Indian population, the *CYP3A53/3* genotype is the most prevalent (46%), and the CYP3A5*3 allele has the highest frequency (65%) [109]. Careful dose adjustments and monitoring are necessary to balance efficacy and safety in this population. On the other hand, DPP‐4 inhibitors may be more effective in lowering glucose levels among Asians, potentially due to their lower BMI, suggesting that these medications could be a more suitable treatment option for Asians compared to other ethnic groups [110, 111]. Hispanic and Indigenous populations display a more diverse genetic background, with varying frequencies of *CYP3A5* expressers and non‐expressers depending on their ancestry [112]. This genetic variability may contribute to differences in drug metabolism rates, necessitating individualised treatment approaches based on pharmacogenetic testing.

The clinical implications of *CYP3A4/5* polymorphisms in diabetes management underscore the need for personalised medicine. Dosing adjustments may be necessary, with African patients often requiring higher doses due to faster metabolism, while European and Asian patients may need lower doses to prevent drug toxicity. Pharmacogenetic testing can play a crucial role in guiding personalised treatment strategies, ensuring that antidiabetic therapy is both effective and safe for diverse populations. Furthermore, recognising these genetic variations can help prevent adverse drug reactions, such as hypoglycaemia in slow metabolizers and suboptimal drug response in fast metabolizers. In conclusion, *CYP3A4/5* polymorphisms significantly influence drug metabolism in diabetic patients, with ethnic differences playing a key role in treatment outcomes. Integrating pharmacogenetic testing into clinical practice can enhance individualised therapy, optimising drug efficacy while minimising the risk of adverse effects across different ethnic groups.

### 5.3 CYP2D6

While *CYP2D6* is not a primary enzyme in the classic T2DM drug metabolism pathway, its omission from pharmacogenetic discussions represents a gap. *CYP2D6* contributes to the metabolism of several medications commonly co‐prescribed in T2DM patients, including antidepressants, beta‐blockers, and antipsychotic agents that can influence weight gain, insulin sensitivity, and adherence [113, 114]. Gene–drug–drug interactions involving *CYP2D6* may therefore indirectly affect glycaemic control and cardiometabolic risk, particularly in patients with multimorbidity.

Moreover, *CYP2D6* polymorphisms may interact with lifestyle factors and polypharmacy, amplifying interindividual variability in treatment response. For example, altered clearance of antidepressants may exacerbate weight gain, appetite changes, or sedation, thereby worsening insulin resistance or reducing adherence to diabetes therapy [115]. Similarly, β‐blockers with excessive plasma exposure in poor metabolizers may mask hypoglycaemia symptoms, increasing clinical risk [116]. These interactions highlight the importance of incorporating *CYP2D6* status into medication selection and monitoring strategies in patients with T2DM.

From a clinical perspective, *CYP2D6* polymorphisms influence dosing and safety of several medications frequently used in patients with T2DM. Poor metabolizers may experience exaggerated pharmacological effects and toxicity from β‐blockers such as metoprolol and carvedilol, necessitating dose reductions [113, 117, 118]. Conversely, ultrarapid metabolizers, more common in African populations, may require higher doses to achieve therapeutic efficacy. Similarly, tricyclic antidepressants prescribed for diabetic neuropathy may accumulate to toxic levels in poor metabolizers, while ultrarapid metabolizers may experience insufficient analgesic benefit [119].

Despite these well‐established pharmacogenetic effects, direct evidence linking *CYP2D6* genotype to long‐term glycaemic outcomes in T2DM remains limited. Most studies focus on pharmacokinetics or adverse drug reactions rather than clinically meaningful endpoints such as HbA1c reduction, hypoglycaemia incidence, or cardiovascular outcomes [120]. Additionally, *CYP2D6* effects are highly context dependent, influenced by polypharmacy, comorbid conditions, environmental factors, and adherence. As newer antidiabetic therapies such as GLP‐1 receptor agonists and dual incretin agents are not primarily metabolised by *CYP2D6*, the enzyme's relevance lies less in direct glucose lowering and more in holistic, patient‐centred medication management [121]. Overall, *CYP2D6* represents an important modifier of treatment safety and effectiveness in T2DM rather than a determinant of disease risk. Incorporating *CYP2D6* pharmacogenetic information may be particularly valuable in patients with complex comorbidities and extensive polypharmacy, supporting safer and more personalised therapeutic strategies.

## 6 Authors' Critical Perspective and Recommendations

From the above, we have consolidated evidence of genetic implications in both the development and management of diabetes. Unravelling such genetic predisposition to diabetes development and management failure could be instrumental in our strides toward tackling diabetes. Although associated with high cost, genetic testing could reveal populations at high risk of developing diabetes. Furthermore, emerging technologies in gene‐targeted therapies and gene editing, such as CRISPR‐Cas9, hold promise for addressing the genetic basis of T2DM [122]. Although still in experimental stages, these approaches could offer transformative solutions by modifying genes associated with metabolic dysfunction. For example, experimental models have demonstrated the potential of silencing specific genes involved in insulin resistance pathways, paving the way for future innovative treatments [123]. Significant challenges, including polygenic disease architecture, off‐target effects, long‐term safety concerns, ethical considerations, and the feasibility of germline or somatic editing in complex metabolic tissues, currently limit their translational relevance. While preclinical studies have demonstrated proof of concept by modulating genes involved in insulin resistance pathways, these findings should be interpreted cautiously and primarily as a foundation for future research rather than as imminent therapeutic solutions.

CYP450 polymorphisms significantly influence drug metabolism in diabetic patients across different ethnic groups, affecting both drug efficacy and safety. Recognising these genetic variations is essential for optimising treatment strategies, minimising adverse effects, and improving therapeutic outcomes. Personalised medicine approaches, such as pharmacogenetic testing, can help tailor drug therapy to an individual's genetic profile, thereby enhancing diabetes management across diverse populations.

The integration of pharmacogenomics into the management of T2DM opens new avenues for personalised care. Precision dosing based on CYP450 genotyping enables tailored drug selection and dosing strategies that reduce the risk of adverse effects while maximising therapeutic efficacy. Additionally, predictive models, such as polygenic risk scores, combine genetic data with clinical factors to identify high‐risk individuals for early intervention and prevention of diabetes‐related complications.

Emerging data on CYP450 polymorphisms highlight the importance of pharmacogenomics for personalised diabetes management. In particular, genetic variants in *CYP2C9, CYP2C19*, and *CYP2D6*, which are known to influence the metabolism of commonly prescribed antidiabetic agents and frequently co‐prescribed medications, have demonstrated clinical relevance across multiple populations. Pharmacogenetic testing may be especially valuable in populations with a high prevalence of functionally significant alleles, such as CYP2C9 *2/*3 and CYP2C19 *2/*17 in European and Asian populations, and copy‐number variants in *CYP2D6*, which are more common in individuals of African ancestry.

Clinically, CYP450 genotyping may be most informative in patients experiencing unexplained adverse drug reactions, suboptimal glycaemic control despite adherence, or when initiating therapies with narrow therapeutic indices or high interindividual variability in metabolism. Another probable approach could involve considering selective CYP450 modulators that fine‐tune drug metabolism without causing excessive inhibition or induction. Moreover, large‐scale clinical trials evaluating the effects of CYP450 modulators on long‐term diabetes outcomes will provide valuable insights to optimise treatment strategies. Modulating CYP450 enzymes offers a promising approach for diabetes management, enabling enhanced drug efficacy, reduced adverse effects, and personalised treatment regimens. With the increasing focus on precision medicine, incorporating CYP450 modulators and pharmacogenomics into diabetes treatment strategies will likely improve patient outcomes and therapeutic success.

Despite these advances, a critical gap remains in our understanding of genetic contributors to T2DM within African populations. The underrepresentation of African cohorts in global genomic studies limits the applicability of findings and perpetuates disparities in care. This shortfall is likely exacerbated by limited funding, inadequate infrastructure, and a shortage of genomic expertise across many African research institutions. To address this, there is a pressing need to invest in locally driven genetic studies and to strengthen regional research capacity.

Equally important is fostering collaborative frameworks among molecular biologists, epidemiologists, and clinicians to enhance translational research and ensure that genetic insights are meaningfully applied in clinical contexts. Furthermore, the adoption of artificial intelligence (AI) tools and machine learning algorithms can significantly augment predictive modelling. As more diverse genetic and clinical data accumulate, AI‐driven models can be developed to stratify individuals by their risk of developing diabetes or their likelihood of achieving glycaemic control. These innovations hold promise for enabling earlier interventions, improving disease monitoring, and optimising therapeutic decision‐making, particularly in resource‐constrained settings.

In conclusion, the path forward must emphasise inclusive, collaborative, and data‐driven approaches to fully harness the potential of genetics in T2DM management. Only through such concerted efforts can precision medicine truly become a global standard, ensuring equitable and effective care for all populations.

## Author Contributions

**Nokwanda N. Ngcobo:** conceptualization, writing – original draft. **Ntethelelo H. Sibiya:** writing – review and editing, conceptualization.

## Funding

The authors have nothing to report.

## Ethics Statement

The authors have nothing to report.

## Consent

The authors have nothing to report.

## Conflicts of Interest

The authors declare no conflicts of interest.

## Data Availability

The authors have nothing to report.
